# Health Professional Students’ Use of Generative Artificial Intelligence During Clinical Placements: Cross-Sectional Online Survey Study

**DOI:** 10.2196/85243

**Published:** 2026-04-27

**Authors:** Sylvain Kotzki, Calvin Massonnet Turner, Kim Gauthier, Mélanie Minoves, Nicolas Vuillerme

**Affiliations:** 1 LIG SANGRIA, Grenoble INP, CNRS, Univ. Grenoble Alpes, Bureau 19, Centre de Recherche en Santé Intégrée (CReSI), Grenoble, 38000, France, 33 476637119; 2 Faculty of Medicine, Univ. Grenoble Alpes, Grenoble, France; 3 HP2, Inserm, Univ. Grenoble Alpes, Grenoble, France; 4 Department of Pharmacy, CHU Grenoble Alpes, Grenoble, France; 5 Institut Universitaire de France, Paris, France

**Keywords:** generative artificial intelligence, AI use, artificial intelligence, health professional students, clinical placements, medical education, ethics

## Abstract

**Background:**

Generative artificial intelligence (GenAI) is rapidly expanding in higher education and clinical practice. However, its use during clinical placements, where cognitive demands and responsibility for patient care increase, remains insufficiently documented.

**Objective:**

This study aimed to characterize self-reported GenAI use during clinical placements, perceived benefits and risks, and related training and governance needs.

**Methods:**

We conducted a cross-sectional online survey at a French university (July 17 to September 30, 2025). Eligible participants were students in medicine, pharmacy, nursing, midwifery, or physiotherapy who were currently in, or had completed within the past 18 months, a clinical placement. A 61-item questionnaire (comprising closed- and open-ended items) assessed GenAI use, task patterns, perceived benefits or risks, and training or governance needs. A composite index classified self-perceived GenAI maturity as minimal, limited, moderate, or high. Group comparisons used χ^2^ tests; maturity gradients used trend tests.

**Results:**

A total of 388 students responded (n=308, 79.4% women), mainly nursing students (n=217, 55.9%). Overall, 204 (52.6%) students reported using GenAI during clinical placements. Use differed across disciplines (*χ*^2^_4_=10.71; *P*=.03), with lower uptake in midwifery (6/23, 26%; odds ratio 0.30, 95% CI 0.11‐0.77). Adoption increased markedly with self-perceived maturity (minimal: 2/22, 9% vs high: 22/29, 76%; trend *P*<.001). Among the 204 users, the most commonly reported uses were information retrieval (n=159, 77.9%), bibliographic search (n=152, 74.5%), and translation or rephrasing (n=145, 71.1%); patient-facing activities were less frequently reported (eg, patient-document drafting or communication preparation: n=78, 38.2%). Although most users reported never entering direct patient identifiers, 48 (23.5%) reported at least 1 disclosure of patient-identifying information, and 96 (47.1%) reported processing real medical content perceived as anonymized. The most endorsed perceived benefits among the 388 students were documentation support (n=315, 81.2%) and improved access to information (n=266, 68.5%). The most endorsed risks were dependency (n=353, 90.9%), skill erosion (n=329, 84.8%), and confidentiality breaches (n=339, 87.4%). Training needs were highest for ethics or regulatory training (294/378, 77.7%) and a best-practice clinical guide (292/373, 78.3%).

**Conclusions:**

GenAI is already used by a substantial proportion of French students in health professions during clinical placements, predominantly for information and documentation support rather than patient-facing activities. Self-perceived readiness is strongly associated with adoption. Reported disclosures and concurrent concerns about dependency, skill erosion, and confidentiality support the need for structured curricula and clear governance frameworks to enable responsible, patient-centered integration of GenAI into clinical education.

## Introduction

Generative artificial intelligence (GenAI) has entered the global health landscape at an unprecedented pace. The widely cited example of ChatGPT (GPT 3.5), which in 2023 achieved passing scores on all 3 components of the United States Medical Licensing Examination, illustrates the capacity of large language models to engage with complex medical knowledge. Concurrently, adoption among health professional students has expanded rapidly. In the United States, just over half of medical students (52%) reported using ChatGPT for medical school-related tasks, based on a survey of 415 students, with about 1 in 6 doing so on a weekly basis [1]. In Israel, 86% of undergraduate students from medicine, nursing, and allied health professions reported being familiar with ChatGPT [2]. Across Europe, a recent survey of 487 medical students across Germany, Austria, and Switzerland revealed that 38.8% of them reported prior experience with artificial intelligence (AI) chatbots such as ChatGPT [3]. In nursing, an umbrella review emphasized both opportunities and substantial pedagogical, ethical, and organizational challenges in integrating AI into education and practice [4]. Consistently, a qualitative study among undergraduate nursing students found that while GenAI was valued as a supportive learning aid, students also expressed concerns over dependency, weakened critical thinking, and threats to academic integrity [5]. In Italy, nearly all physiotherapy students (≈95.3%) were aware of AI chatbots, though more than half reported never using them in academic settings [6].

From an educational perspective, 3 primary applications of GenAI have been identified: the generation and reformulation of academic content, instant adaptive tutoring, and participation in simulation or problem-based learning scenarios [7-9]. A recent systematic review further synthesized evidence across health professional education, confirming the growing influence of GenAI on student learning, particularly in acquisition, inquiry, practice, and production [10]. Importantly, this growing body of evidence extends to experimental research, with a randomized controlled trial in Norway reporting a modest improvement in knowledge test performance when pharmacy students had access to ChatGPT, although the effect did not reach statistical significance [11]. However, this rapid adoption is unfolding amid a marked institutional vacuum: more than 60% of surveyed pharmacy students reported no formal curricular exposure to GenAI [12], while in 2024, an analysis of 116 US research universities found that less than half provided classroom guidance and many still lacked formal institutional policies [13]. As a result, health professional education is evolving in a state of continuous adaptation, where the promising capabilities of GenAI coexist with substantial risks, including facilitated plagiarism, overreliance, diminished critical thinking, weakened communication, and threats to the development of clinical reasoning and problem-solving skills [14].

Beyond classroom-based learning, the training of health professionals also depends heavily on clinical practice, as their training does not take place solely in lecture halls but also through clinical placements. These clinical placements, while essential to professional development, place students in a dual role: learning in real-world conditions while gradually assuming responsibility for patient care. Such placements are frequently marked by heightened stress, significant emotional demands, feelings of inadequacy [15,16], and in some cases, early signs of burnout [17]. Meanwhile, in clinical practice, GenAI is increasingly being applied across health care settings such as hospitals, clinics, private practices, and pharmacies. Recent reports describe its use to accelerate documentation, simplify radiology reports, draft consultation letters, and even support aspects of diagnostic reasoning [18,19]. An integrative review in nursing similarly highlighted applications of AI across education, clinical practice, workload management, and professional perceptions, pointing to opportunities but also to enduring structural and ethical challenges [20]. In this environment, health professional students in clinical placements are likely to turn to GenAI to ease their daily workload, such as checking information, translating a consent form, or rephrasing a discharge summary. While such assistance can provide immediate support when human feedback is unavailable, it also raises concerns about overreliance, the weakening of critical learning processes, and the potential for errors.

A recent survey found that 1 in 5 UK general practitioners reported using GenAI, most commonly to generate documentation after patient encounters. Clinicians acknowledged its potential to reduce administrative burden but also raised concerns regarding patient safety, data privacy, and the risk of errors [21]. Other authors caution, however, that adoption may remain limited or prove transient in the absence of robust clinical validation [22]. Health professional students in clinical placements stand at the intersection of 3 evolving contexts: higher education, where GenAI is rapidly becoming mainstream; clinical environments, where health care professionals are beginning to adopt it; and a learning experience often marked by uncertainty, stress, and substantial cognitive demands. Yet, to the best of our knowledge, no survey has systematically examined how students use GenAI during clinical placements, what motivates their engagement, and the perceived benefits, risks, and governance needs associated with this pivotal stage of professional development.

To address this gap, we conducted an anonymous survey among 388 health professional students from multiple health care programs at the Université Grenoble Alpes (UGA, France), all of whom had recently completed or were currently engaged in clinical placements. The aim of this study was to characterize health professional students’ self-reported use of generative AI in clinical situations. Specifically, we sought to (1) estimate adoption prevalence overall and by discipline, (2) describe the tasks, tools, and frequency of use, including self-reported handling of patient-related information, and (3) assess perceived benefits, risks, and training and governance needs to inform structured educational and institutional responses.

## Methods

### Study Design

We conducted a web-based descriptive cross-sectional study in the form of an online survey targeting health professional students at UGA (France). The aim was to document actual use, perceived benefits and risks, as well as training needs related to GenAI in the context of clinical placements. Data collection took place between July 17 and September 30, 2025. This survey was carried out as part of a national initiative, funded by the French National Research Agency, to support the development and dissemination of digital health and AI in health education in French university health programs (France 2030 program, ANR n°23-CMAS-0035).

### Ethical Considerations

This study has received ethical approval from the “Ethics Committee for the Integrity and Ethics of Research in Health Professions Education” of the “*Société Internationale Francophone d’Education Médicale*” (Notice n°1905‐2025, issued July 17, 2025). Entry into the survey was conditional upon acceptance of an electronic informed consent form, accompanied by an information sheet detailing the study objectives, data confidentiality, voluntary nature of participation, right to withdraw, and potential risks. The survey was fully anonymous, and no incentives were offered for participation. Participation was voluntary, and informed consent was obtained electronically from all participants.

### Recruitment Procedure

Following approval by the deans of the Faculties of Medicine and Pharmacy at UGA, an email invitation via the official academic mailing list was distributed to potential participants enrolled in medical or allied health training programs. Overall, approximately 4553 eligible students (n=2196 in medicine, n=283 in pharmacy, n=1904 in nursing, n=120 in midwifery, and n=50 in physiotherapy) were invited (see Multimedia Appendix 1 for details). In addition, a flyer was posted on student information boards at the entrance of lecture halls and classrooms throughout the recruitment period, ensuring a reinforced exposure to the survey. A total of 388 students completed the survey, corresponding to an overall response rate of 8.5%, ranging from 3.1% in medicine to 26% in physiotherapy. Interested students accessed the survey via a LimeSurvey link.

### Clinical Placement Context

In the French health professions education system, clinical placements are mandatory components of curricula. Rotations typically last 2 to 10 weeks. Students progressively assume responsibilities according to their year of study, under the supervision of licensed health professionals from the relevant discipline, with day-to-day oversight and validation of key care and documentation tasks. Early-year students mainly observe and perform basic tasks, while senior students (in the final years of training) take a more active role in discipline-specific activities, including patient care tasks, documentation, information-seeking, care coordination, and patient communication or education.

### Data Collection

We collected data through an anonymous online survey administered with LimeSurvey. Students interested in the study were invited to access the anonymous link, which contained 61 items: 18 single-choice questions, 38 single-choice questions on a 4-point Likert scale (1=strongly disagree to 4=strongly agree), 1 multiple-choice question, and 4 open-ended questions. The full survey questionnaire is available in Multimedia Appendix 1.

The items were developed based on 4 published studies: Blease et al [21] on generative GenAI in primary care, Lobet et al [23] on the spontaneous use of ChatGPT by university students, Karaca et al [24] on the Medical Artificial Intelligence Readiness Scale for Medical Students, and Boillat et al [25] on physician and student preparedness for AI. Items were then adapted by consensus to the clinical context.

We organized the instrument into 4 domains:

- Sociodemographic characteristics
- Declared uses of GenAI in clinical practice
- Perceptions and attitudes in clinical contexts
- Knowledge and training related to GenAI

We conducted this web-based survey in accordance with the CHERRIES (Checklist for Reporting Results of Internet E-Surveys) checklist (Checklist 1) [26]. The questionnaire was hosted on LimeSurvey and included a single skip logic based on declared GenAI use versus nonuse; all other sections were shown to all participants. A total of 38 items were mandatory; missing responses were otherwise allowed, and respondents could review or change answers before submission. Only fully submitted questionnaires were analyzed, and no technical measures (cookies, IP checks, or registration) were used to prevent multiple entries.

Content validity was assessed through expert review by 2 clinicians and health-professions education specialists (SK and MM), who evaluated item relevance, clarity, and coverage of the 4 intended domains. Minor wording changes were made to improve clarity and contextual alignment with clinical placements (4 items required rewording for better understanding; no items were removed or added). A pilot test with 10 students (n=4 students in medicine, n=3 students in pharmacy, and n=3 students in nursing) confirmed intelligibility. Feedback from experts and students was incorporated to produce the definitive version. The average completion time was 15 minutes.

### GenAI Maturity Index

To capture students’ self-perceived maturity toward GenAI, considered as their perceived readiness to understand, evaluate, and appropriately use GenAI in educational and professional contexts, we constructed a composite GenAI maturity index from 9 Likert-type items [24]. This approach follows previous work on AI readiness and familiarity in health professions, where multiple items are aggregated into a single readiness or familiarity score [24,25]. One item assessed general digital comfort (“I feel comfortable with digital technologies in general”), and 8 items targeted GenAI-specific knowledge and self-efficacy (basic GenAI concepts, distinction between GenAI and other AI, ability to judge the reliability of GenAI outputs, awareness of appropriate use contexts and current limitations, and perceived ability to use GenAI in one’s education and future practice (full wording in Multimedia Appendix 1).

All 9 items used a 4-point agreement scale (1=strongly disagree, 4=strongly agree). To respect the ordinal nature of the 4-point Likert responses, we computed, for each respondent, the median of the 9 item scores and rounded it down to the nearest integer. This yielded a 4-level ordinal GenAI maturity score: 1 (minimal), 2 (limited), 3 (moderate), and 4 (high maturity). We required at least 5 nonmissing responses out of 9 (≥50%) to compute the index, consistent with commonly used “half-rule” approaches for scoring multi-item scales in the presence of item-level missingness [27].

In this sample, the 9 items showed good internal consistency (Cronbach *α*=0.869). Internal consistency was similar when excluding the general digital-comfort item (*α*=0.870 for items 2‐9), suggesting that digital comfort functions as a foundational component of the same underlying construct. Inspired by previous AI-readiness instruments that combine several facets into a single readiness score [24,25], we therefore use this composite GenAI maturity index pragmatically as an ordinal stratification of self-perceived GenAI maturity.

### Qualitative Analysis of Open-Ended Responses

Open-ended responses were analyzed using a qualitative descriptive approach with descriptive content analysis. Responses were segmented into 213 meaning units (eg, items separated by semicolons or distinct ideas) and analyzed by question (reasons for nonuse, additional tasks, suggested uses, suggested risks, and training or support needs). A hybrid coding strategy was used, combining a first deductive framework aligned with these domains and inductive identification of recurrent subthemes. Two researchers (SK and MM) independently coded all segments using the agreed codebook. Initial agreement was 83.1% (177/213); disagreements (36/213, 16.9%) were resolved through discussion until consensus, and the final coding was used for thematic reporting and choice of illustrative quotations. Quotations were anonymized and translated from French to English. Qualitative findings are reported as recurring themes with illustrative excerpts to complement the quantitative results rather than as a stand-alone qualitative study.

### Data Processing and Statistical Analyses

Data were exported to Excel and analyzed in Python (version 3.11) using pandas (2.2.2), NumPy (1.26.4), SciPy (1.13.1), statsmodels (0.14.2), and matplotlib (3.9.2). Quantitative variables were described using means or medians with IQRs, while qualitative variables were presented as counts and percentages.

We performed overall group comparisons using Pearson chi-square test to assess the heterogeneity of distributions across academic disciplines and maturity levels. When expected cell counts were small, Fisher exact test was applied. For pairwise comparisons between a given discipline and all others, odds ratios with 95% CIs were calculated.

To assess gradients across maturity levels, 4-point Likert responses were dichotomized into 2 categories (disagree=1‐2; agree=3‐4) to facilitate interpretation and to enable proportion-based trend testing across ordered groups [28-30]. Linear trends were then assessed using the Cochran-Armitage trend test. When multiple simultaneous comparisons were conducted, the family-wise error rate was controlled using the Holm method. Statistical significance was set at *P* less than .05. We acknowledge that dichotomizing ordinal responses entails information loss compared with ordinal models [28].

## Results

### Respondent Demographics and Academic Profile

The survey included 388 health professional students (Table 1), predominantly women (n=308, 79.4%), with most respondents aged 20‐24 years (n=198, 51.0%). The sample was largely composed of nursing students (n=217, 55.9%), with additional representation from medicine, pharmacy, midwifery, and physiotherapy programs (Table 1). Years of study covered the full curriculum, with a higher proportion of early-year students (y 1‐2) and smaller proportions in later years (Table 1).

Among the 388 respondents, 380 (97.9%) provided sufficient data to compute the GenAI maturity index. Most students reported limited to moderate maturity (levels 2‐3: 329/380, 86.6%), while relatively few reported minimal or high maturity (levels 1 and 4: 51/380, 13.4%; Table 1). Item-level details are provided in Multimedia Appendix 1.

**Table 1.** Descriptive table of responder characteristics (n=388).

Characteristics	Participants, n (%)
Gender	
Man	76 (19.6)
Woman	308 (79.4)
Prefer not to answer	4 (1.0)
Age (y)	
<20	55 (14.2)
20‐24	198 (51.0)
25‐29	55 (14.2)
30‐34	22 (5.7)
>34	58 (15.0)
Disciplines	
Medicine	69 (17.8)
Midwifery	23 (5.9)
Nursing	217 (55.9)
Pharmacy	66 (17.0)
Physiotherapy	13 (3.4)
Year of study	
First year	82 (21.1)
Second year	97 (25.0)
Third year	77 (19.9)
Fourth year	29 (7.5)
Fifth year	52 (13.4)
Sixth year	10 (2.6)
Beyond the sixth year	41 (10.6)
Self-reported GenAI^a^ maturity	
Minimal (1)	22 (5.7)
Limited (2)	130 (33.5)
Moderate (3)	199 (51.3)
High (4)	29 (7.5)
NA^b^	8 (2.1)

a GenAI: generative artificial intelligence.

b NA: not applicable.

### Self-Reported Uses

#### GenAI Use in Clinical Placement

Overall, 204 of 388 (52.6%) students reported using GenAI during clinical placements (Table 2). Adoption differed across disciplines (χ^2^_4_=10.71; *P*=.03), primarily reflecting lower uptake among midwifery students (odds ratio 0.30, 95% CI 0.11‐0.77; Holm-adjusted *P*=.049). Pharmacy students showed the highest unadjusted proportion of use, but this difference was not statistically significant after correction (Table 2). GenAI use increased markedly with higher self-perceived GenAI maturity, from 2 of 22 (9%) in the minimal group to 22 of 29 (76%) in the high-maturity group (trend test *z* score=6.01, *P*<.001; Table 3).

**Table 2.** Prevalence of GenAI^a^ use in clinical placements by discipline.

	Yes, n (%)	No, n (%)	OR^b^ (95% CI) vs rest	*P* value
GenAI use in clinical placements				
Overall	204 (52.6)	184 (47.4)	—^c^	—
GenAI use by discipline				
Medicine	33 (47.8)	36 (52.2)	0.79 (0.47-1.34)	.99
Midwifery	6 (26.1)	17 (73.9)	0.3 (0.11-0.77)	.049^d^
Nursing	117 (53.9)	100 (46.1)	1.13 (0.76-1.69)	.99
Pharmacy	42 (63.6)	24 (36.4)	1.73 (1.00-2.99)	.23
Physiotherapy	6 (46.1)	7 (53.9)	0.77 (0.25-2.32)	.78

a GenAI: generative artificial intelligence.

b OR: odds ratio.

c Not applicable.

d Statistically significant after Holm adjustment.

**Table 3.** Prevalence of GenAI^a^ use in clinical placements by self-perceived GenAI maturity.

GenAI use by self-perceived GenAI maturity	Yes, n (%)	No, n (%)	*χ*^2^ Pearson (*df*; *P* value)	Cochran-Armitage trend: *z* score (*P* value)
Maturity score			37.25 (3; <.001)	6.01 (<.001)
Minimal (1)	2 (9.1)	20 (90.9)		
Limited (2)	52 (40.0)	78 (60.0)		
Moderate (3)	122 (61.3)	77 (38.7)		
High (4)	22 (75.9)	7 (24.1)		

a GenAI: generative artificial intelligence.

#### Reasons for Not Adopting GenAI During Clinical Placements Situations

Among nonusers (184/388), the most often reported reasons were not perceiving a need (59/184, 32.1%), not knowing best practices for use (38/184, 20.7%), and lack of interest (34/184, 18.5%; Table 4). Fewer respondents reported not knowing how to use the tools (18/184, 9.8%) or not being aware of them (10/184, 5.4%); “Other” was selected by 25/184 (13.6%).

**Table 4.** Reasons for not adopting generative artificial intelligence during clinical placements among nonuser respondents (n=184).

Reason for nonuse	Overall, n (%)	Nursing, n (%)	Medicine, n (%)	Pharmacy, n (%)	Midwifery, n (%)	Physio, n (%)
It does not interest me	34 (18.5)	13 (13)	10 (27.8)	3 (12.5)	5 (29.4)	3 (42.9)
I do not need it	59 (32.1)	32 (32)	10 (27.8)	9 (37.5)	6 (35.3)	2 (28.6)
I do not know how to use it	18 (9.8)	12 (12)	4 (11.1)	2 (8.3)	0 (0)	0 (0)
I don’t know the best practices for using it	38 (20.7)	25 (25)	7 (19.4)	2 (8.3)	3 (17.6)	1 (14.3)
I was not aware of it	10 (5.4)	6 (6)	0 (0)	3 (12.5)	1 (5.9)	0 (0)
Other	25 (13.6)	12 (12)	5 (13.9)	5 (20.8)	2 (11.8)	1 (14.3)

Reasons varied across disciplines, but subgroup sizes were small for some programs. Overall, responses suggested two main profiles: (1) attitudinal barriers (eg, no perceived need or lack of interest) and (2) guidance-related barriers (eg, lack of best practices or technical know-how), with their relative weight differing by discipline (Table 4).

Open-ended responses under “Other” (29 meaning units) mostly referred to perceived unreliability and risk of errors, environmental impact, or broader socio-technical critique, and a preference for official sources or local protocols over GenAI. The full thematic breakdown with illustrative quotations is provided in Multimedia Appendix 1.

#### Self-Reported Use of GenAI During Clinical Placements Situations

Among GenAI users (204/388), reported use was primarily oriented toward information-related and documentation tasks (Table 5). The most common uses included information extraction (159/204, 77.9%) and bibliographic search (152/204, 74.5%), while patient-facing activities were less frequently reported (eg, drafting patient documents or preparing patient communication: 78/204, 38.2% each; Table 5). This pattern was mirrored in usage frequency: weekly-or-more use was more common for information-related tasks (eg, information extraction: 53/204, 26.0%) than for patient-facing documentation or communication (13/204, 6.4% and 15/204, 7.4%; Table 5).

Free-text comments on additional tasks (106 meaning units) most often described learning support or pedagogical explanations and writing or text revision; the full thematic breakdown with illustrative quotations is provided in Multimedia Appendix 1.

**Table 5.** Frequency of generative artificial intelligence usage by task (n=204).

Item	Ever used, n (%)	At least once a week, n (%)
Content translation	145 (71.1)	19 (9.3)
Report or article writing	141 (69.1)	27 (13.2)
Info extraction	159 (77.9)	53 (26)
Bibliographic search	152 (74.5)	47 (23)
Clinical simulation	112 (54.9)	31 (15.2)
Drafting patient documents	78 (38.2)	13 (6.4)
Preparing patient communication	78 (38.2)	15 (7.4)

#### Disclosure of Sensitive Information to GenAI

Among GenAI users, most reported that entering direct patient identifiers had “never happened” (151/204, 74.0%; Table 6). However, 48 of 204 (23.5%) students reported at least 1 instance (once, a few times, often, or unintentionally), 5 (2.5%) were unsure, and only 3 (1.5%) reported that this occurs “often.”

Self-disclosure was more common. While 109 of 204 (53.4%) students reported “never” entering their own personal identifiers, 87 (42.6%) reported at least 1 instance, 8 (3.9%) were unsure, and 18 (8.8%) reported that this occurs “often” (Table 6). The broadest category of disclosures involved real medical content: 100 (49%) reported “never,” whereas 96 (47.1%) reported processing real medical data (even if perceived as anonymized), including 20 (9.8%) reporting “often” (Table 6).

**Table 6.** Self-reported disclosure of sensitive information when using generative artificial intelligence (n=204).

Type of data disclosed	Never, n (%)	Once, n (%)	A few times, n (%)	Often, n (%)	Maybe^a^, n (%)	Do not know, n (%)
Patient-identifying data (*name, ID, address*)	151 (74)	12 (5.9)	22 (10.8)	3 (1.5)	11 (5.4)	5 (2.5)
Student’s own personal data (*name, identifiers*)	109 (53.4)	18 (8.8)	39 (19.1)	18 (8.8)	12 (5.9)	8 (3.9)
Real medical data (*anonymized or not*)	100 (49.0)	17 (8.3)	56 (27.5)	20 (9.8)	3 (1.5)	8 (3.9)

a It may have happened unintentionally (eg, by copying and pasting).

### Perceived Benefits and Risks

#### Perceived Benefits of GenAI Use in Clinical Practice

**Figure 1.**
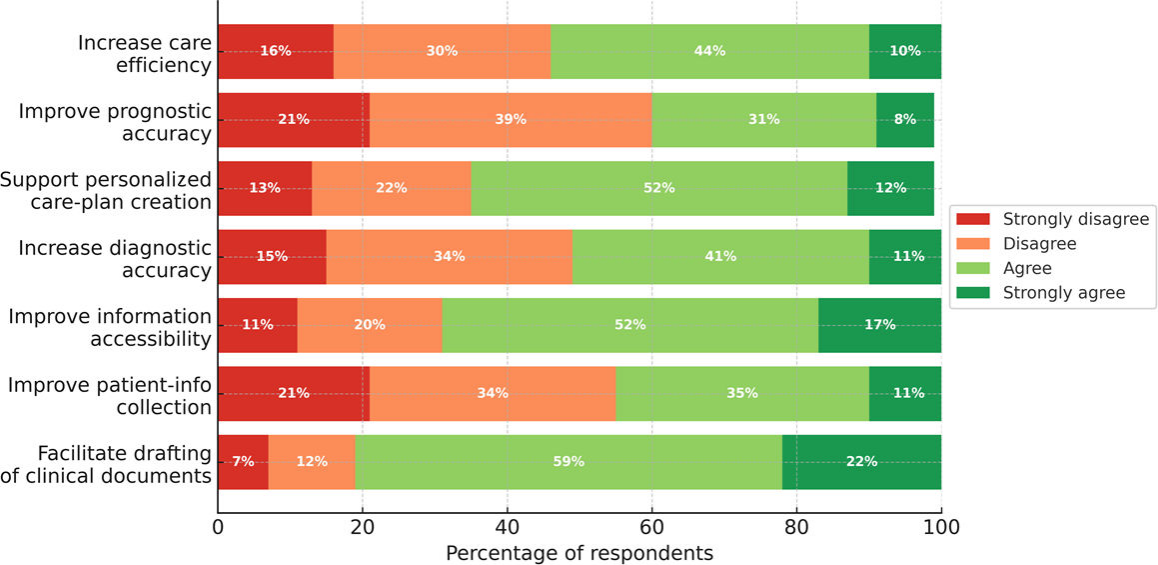
Perceived benefits of generative artificial intelligence for clinical practice during placements (n=388).

Students rated seven potential benefits of GenAI in clinical practice on a 4-point Likert scale (n=388; Figure 1). Overall, perceived benefits were highest for facilitating the drafting of clinical documents (315/388, 81.2% agree or strongly agree) and improving information accessibility (266/388, 68.5%). Intermediate endorsement was observed for supporting personalized care plan creation, increasing care efficiency, and improving diagnostic accuracy (Figure 1). In contrast, views were more skeptical regarding patient-information collection and prognostic accuracy, for which most respondents disagreed (212/388, 54.6% and 234/388, 60.3%, respectively). Medians indicated overall agreement for 5 of 7 potential benefits, whereas patient information collection and prognostic accuracy had median disagreement. Full response distributions, item-level medians, and IQRs are reported in Multimedia Appendix 1.

Free-text proposals about potential clinical uses were segmented into 22 coded meaning units and were consistent with the quantitative patterns. The most frequent themes concerned reflective support and systematic checklists and administrative or documentation streamlining. The full thematic breakdown, including less frequent themes and illustrative quotations, is provided in Multimedia Appendix 1.

#### Perceived Risks of GenAI Use in Clinical Practice

**Figure 2.**
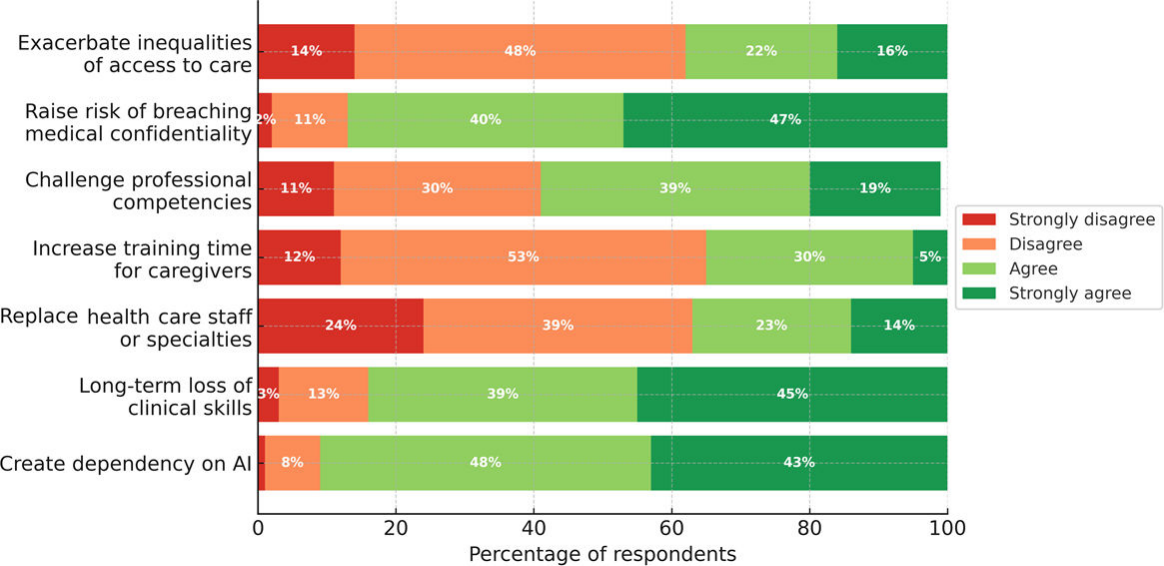
Perceived risks of clinical use of generative artificial intelligence during placements (n=388). AI: artificial intelligence.

Students rated seven potential risks of GenAI use in clinical practice on a 4-point Likert scale (n=388; Figure 2). Three concerns were strongly endorsed: creating dependency on GenAI (353/388, 90.9% agree or strongly agree), long-term loss of clinical skills (329/388, 84.8%), and breaching medical confidentiality (339/388, 87.4%). A more moderate majority agreed that GenAI could challenge professional competencies (228/388, 58.8%; Figure 2). In contrast, most respondents disagreed that GenAI would replace health care staff or specialists, increase training time for caregivers, or exacerbate inequalities in access to care (all >60% disagree or strongly disagree; Figure 2). Medians showed agreement for 4 of 7 risks, while replacement of staff, increased training time, and inequalities had median disagreement. Full response distributions, item-level medians, and IQRs are reported in Multimedia Appendix 1.

Open-ended risk comments were segmented into 32 coded meaning units and were consistent with the hierarchy seen in the Likert ratings. The most frequent themes concerned risks of errors and potential patient-safety consequences, dependence or reduced reflective capacity, and relational impacts (eg, depersonalization or reduced quality of the clinician-patient relationship). The full thematic breakdown, including less frequent themes and illustrative quotations, is provided in Multimedia Appendix 1.

### Training and Support Needs for GenAI Use

**Figure 3.**
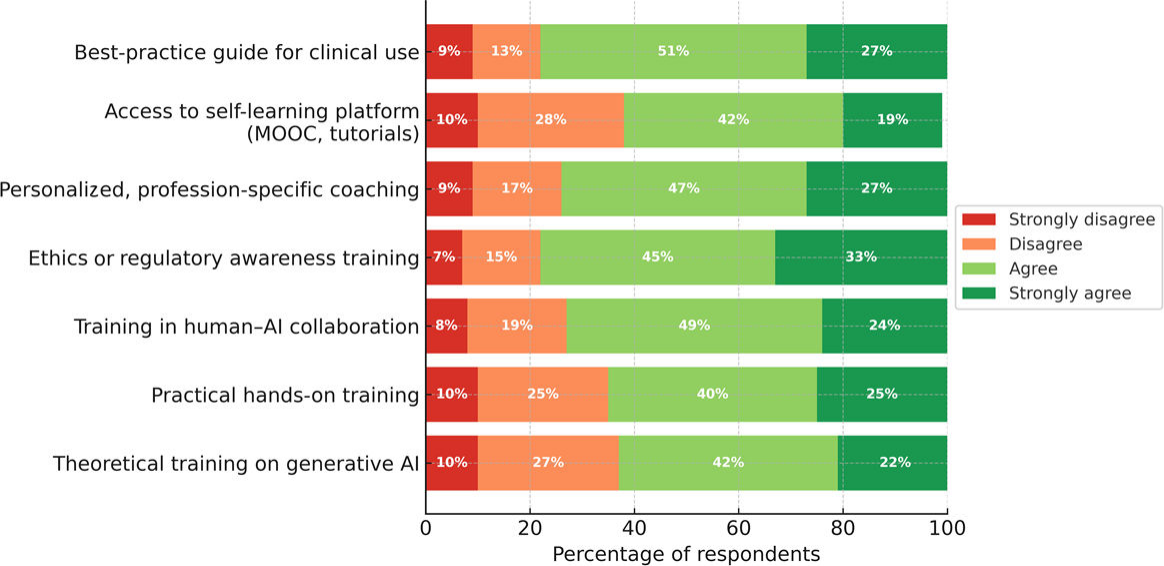
Training and support needs for generative artificial intelligence (n=388). AI: artificial intelligence; MOOC: massive open online course.

Across 7 items assessing training and support needs, the median response was “Agree” for every statement (Figure 3). The strongest endorsement concerned a best-practice clinical guide (292/373, 78.3% agree or strongly agree) and ethics or regulatory awareness training (294/378, 77.7%). Important levels of agreement were also observed for profession-specific coaching and training in human-AI collaboration (Figure 3). Overall disagreement remained limited (strongly disagree ≤11% across items). Full response distributions, item-level medians, and IQRs are reported in Multimedia Appendix 1.

Free-text comments on additional needs (24 meaning units) provided further context. A large share reported no additional needs or interest (8/24, 33%). Among expressed needs, the most frequent theme concerned practical training and ongoing support to integrate GenAI appropriately into clinical learning and practice (6/24, 25%). The full thematic breakdown, including less frequent themes and illustrative quotations, is provided in Multimedia Appendix 1.

### Governance and Organizational Readiness

**Figure 4.**
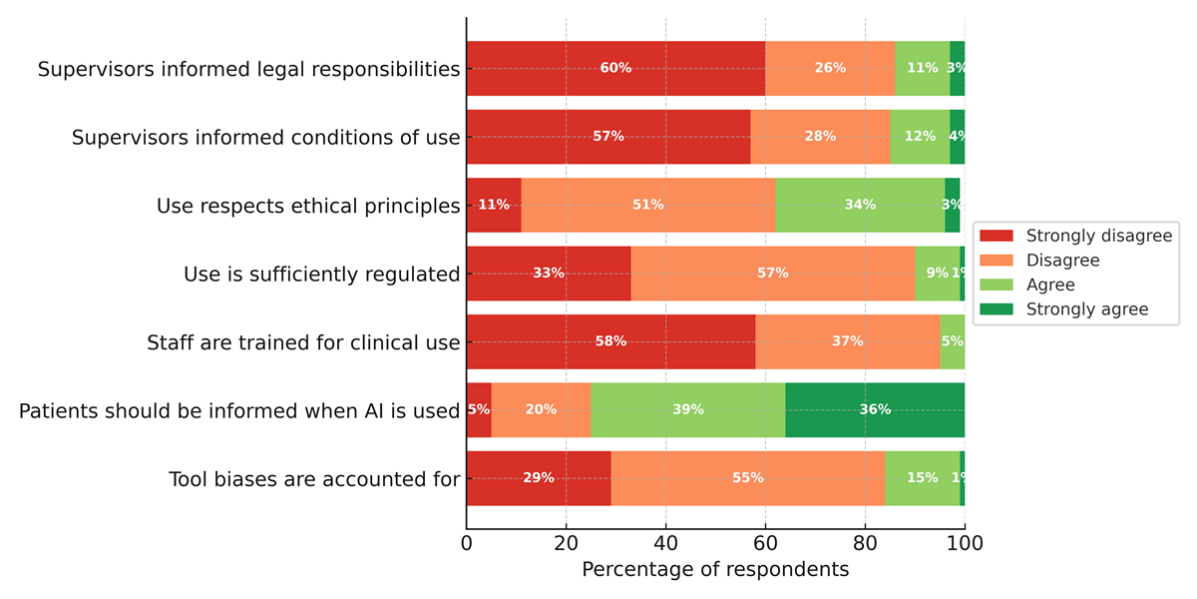
Governance and organizational readiness (n=388). AI: artificial intelligence.

Seven items assessed students’ perceptions of organizational readiness and governance for GenAI use during clinical placements (Figure 4). Overall, respondents perceived limited institutional communication and safeguards: only a small minority recalled supervisors providing information on legal responsibilities or local conditions of use (Figure 4). Governance was also perceived as insufficient, with 36 of 359 (10%) agreeing that GenAI use is sufficiently regulated. Operational readiness received the poorest ratings, as only 18 of 372 (4.8%) agreed that staff are trained for clinical use and 56 of 343 (16.3%) agreed that tool biases are accounted for. In contrast, transparency toward patients appeared as the single area of strong consensus: 248 of 331 (74.9%) agreed that patients should be informed when AI is used in their care (Figure 4). Full response distributions, item-level medians, and IQRs are reported in Multimedia Appendix 1.

## Discussion

### Principal Findings

In this cross-sectional survey of 388 French students in health professions, 52.6% (204/388) reported using GenAI during clinical placements. Adoption varied across disciplines, with lower uptake in midwifery, and increased markedly with higher self-perceived GenAI maturity. Reported uses primarily involved information retrieval and documentation- or writing-related tasks, while patient-facing activities were less frequently reported.

Notably, close to a quarter of GenAI users reported at least 1 instance of entering patient-identifying information, and close to half reported processing real medical content perceived as anonymized. Students perceived substantial benefits for documentation support but also expressed prominent concerns about dependency, erosion of clinical skills, and breaches of confidentiality, alongside strong demand for ethics or regulation training and best-practice guidance. Overall, these findings show that GenAI is already embedded in students’ placement practices, but with uneven adoption, limited institutional framing, and persistent confidentiality risks.

### Learning to Think With GenAI During Clinical Placements

In this survey, students appeared to mobilize GenAI during placements primarily as a cognitive and organizational aid (eg, information retrieval and documentation support) rather than as a tool embedded in direct patient-facing work. This “low-stakes *versus* clinically situated” pattern plausibly reflects implicit boundaries around what feels safe, acceptable, and professionally legitimate to delegate to a third-party model in real clinical environments and aligns with students’ expressed need for clear best-practice guidance.

Perceived readiness offers an additional lens to interpret why some students integrate GenAI into placement work while others do not. Our maturity index should be understood as a pragmatic stratification of how prepared students feel to evaluate and use GenAI critically in clinical learning contexts, rather than as an objective measure of competence. The strong adoption gradient across readiness levels is consistent with AI-readiness frameworks in health professions education [24,25]. Emerging evidence also suggests that structured GenAI-supported simulations can improve targeted competencies in medical students [31,32]. Educationally, this matters because uneven readiness may translate into uneven exposure to GenAI practices during placements, potentially reinforcing a hidden curriculum in which norms of safe and legitimate use are acquired informally and shape professional identity formation at a formative stage [33,34].

This raises a broader tension between efficiency and reflection in workplace learning. While GenAI may support information management and autonomous learning [35], medical documentation and reasoning processes are also critical spaces for cultivating clinical rigor, accountability, and reflective judgment. Without explicit pedagogical framing, early delegation of reasoning-related tasks may narrow opportunities for reflexivity and increase vulnerability to automation bias and overreliance. These mechanisms were previously described in clinical decision support contexts, where they can compromise patient safety [36]. Conversely, when embedded in structured educational approaches (eg, simulation with feedback and validated rubrics), GenAI can be leveraged to enrich reflection and support deliberate practice, as illustrated by initiatives such as MedSimAI [32]. Collectively, these findings support the need to move from informal, individual experimentation toward supervised and pedagogically grounded uses of GenAI in clinical learning environments [34,36].

### Interpreting Nonuse, Perceived Benefits, and Perceived Risks

Students’ reasons for not using GenAI during placements suggested that nonadoption often reflected deliberate reservations rather than simple unfamiliarity. Reported concerns included limited perceived clinical relevance, fear of diminished reflexivity, confidentiality risks, and broader socio-technical critiques, such as environmental impact. This aligns with Tortella et al [6], who observed that reluctance among physiotherapy students was largely driven by concerns about inaccuracy and error, and perceived limits for complex tasks, rather than a lack of awareness. From a teaching perspective, such reservations should be treated as a pedagogical resource: they create opportunities to make expectations explicit and to cultivate critical judgment, irrespective of whether students ultimately adopt GenAI tools.

Perceived benefits were large but not uniform. Students strongly endorsed GenAI for documentation relief and improved access to information, positioning it primarily as a cognitive and organizational facilitator. In contrast, endorsement was more cautious for higher-stakes functions such as diagnostic support, personalized planning, and efficiency gains, reflecting conditional trust rather than full reliance. This pattern is consistent with Janumpally et al [37], who emphasized that GenAI may support learning and selected reasoning tasks but remains constrained by output reliability and cannot substitute for human judgment, particularly in domains directly implicating professional responsibility, such as patient data collection and prognostication.

Perceived risks were dominated by concerns about dependency, long-term erosion of clinical skills, and confidentiality breaches, alongside worries about bias, uncertain legal accountability, and a more subtle displacement of professional responsibility and human interaction. These concerns resonate with the broader literature on automation bias and overreliance in clinical decision support, including discussions of “assistive AI” as the lowest level of autonomy that may still erode vigilance if not framed and supervised [36]. Importantly, students did not primarily express fears of being replaced. Rather, they emphasized risks of weakened judgment, diluted accountability, and reduced quality of the care relationship. These concerns suggest that GenAI integration in workplace learning should be structured, reflective, and explicitly governed rather than left to informal experimentation [6,36,37].

### Confidentiality and Data Governance

From a patient-safety and governance perspective, the disclosure of sensitive information calls for specific attention. In our sample, 23.5% (48/204) reported at least 1 instance of entering patient-identifying information, and 47.1% (96/204) reported processing real medical content perceived as anonymized. Even when students perceive data as anonymized, free-text prompts may contain contextual details that increase reidentification risk, and the use of nonapproved third-party tools may involve data processing outside institutional control (eg, storage and transfer conditions) [38,39]. In European contexts, this directly intersects with General Data Protection Regulation obligations and institutional requirements for handling health data [40].

These findings imply a need for explicit supervision and coordinated governance during placements. Placement supervisors, faculty, and host clinical sites (eg, hospital departments and private practices) should clearly define acceptable versus unacceptable prompting, provide concrete deidentification rules and examples, and ensure access to approved, compliant tools (or clear prohibitions where such tools are not available). Because clinical training occurs across multiple host sites, governance should be coordinated between the academic institution and placement providers so that expectations and safeguards are consistent across settings. At the institutional level, local charters and “allowed tool” policies aligned with data-protection governance should go with training, so that confidentiality norms are not left to informal experimentation. In France, this approach is consistent with recent institutional guidance: the French National Academy of Medicine discusses ethical issues and recommendations for the use of GenAI in health [41], and the French National Authority for Health has published a practical guide to support appropriate and informed use of GenAI by health professionals [42].

### From Technical Literacy to Patient-Centered GenAI Education

Students’ expectations for training extend beyond technical mastery toward the full triad of knowledge, skills, and professional attitudes: knowledge of ethical and regulatory frameworks; practical competencies developed through workshops and profession-specific coaching; and professional dispositions cultivated through mentorship, reflexivity, and attention to sustainability. This aligns with calls to design health AI curricula that move past technical literacy to incorporate critical thinking, ethics, and patient-centered judgment. Komasawa and Yokohira [43] argue that the rise of GenAI requires rethinking health professionalism by combining technological fluency with enduring humanistic values such as empathy, integrity, and accountability.

### A Pragmatic Pathway From Informal Adoption to Responsible Integration

Our findings point to an institutional readiness gap during placements: limited shared benchmarks, uneven supervisory guidance, and insufficient attention to bias, regulation, and data governance. They also support a stepwise pathway to move from informal, student-driven use toward safe and educationally meaningful integration across disciplines. First, a transversal common core should address ethics, regulation, professional standards, and model limitations or uncertainty, alongside concrete rules for health data handling in GenAI contexts (what must never be entered, how to deidentify, and which tools are approved). Second, competency development requires practice beyond theory: simulation- or case-based activities with GenAI, explicit reflection prompts, and structured feedback can help students learn when GenAI supports reasoning versus when it undermines it (eg, automation bias and overreliance) and can contribute to professional identity formation. Third, training for both academic staff and host-site supervisors is essential so that placement supervisors can set shared expectations, mentor reflective use, and intervene when unsafe practices occur. Fourth, institutional governance should provide clear local policies (eg, charters, approved tool lists, patient transparency where relevant, and escalation procedures for data incidents), ensuring alignment between pedagogy and professional standards. This enabling framework does not mandate adoption; it operationalizes conditions under which GenAI use remains compatible with reflexivity, professional accountability, and patient-centered care, and can help transform spontaneous use into integrated, supervised competencies in clinical learning settings, as illustrated by structured simulation initiatives such as MedSimAI [32].

### Limitations

This study has several limitations. First, it was conducted within a single French university (UGA), which limits international generalizability. The survey was embedded in a national initiative funded by the French National Research Agency to support the rollout of digital health and AI in health education across French university training programs. While this may strengthen the transferability of educational implications, it does not eliminate the single-site constraint. Notably, the main trends observed here are consistent with the international survey by Busch et al [44] (4596 students from 192 faculties across 48 countries), which similarly reported widespread student adoption of generative tools alongside a global deficit in formal AI education. Second, GenAI use and data-disclosure behaviors were measured through self-report and may be influenced by recall and social desirability biases. Although responses were collected anonymously and without incentives, self-reporting remains susceptible to these biases. Nevertheless, documenting students’ lived practices and perceptions was a core aim, particularly in contexts where formal pedagogical frameworks are still emerging; as such, perceptions may be viewed as outcomes in their own right, consistent with Busch et al [44]. Third, we did not collect data from clinical supervisors or educational leaders and therefore cannot characterize institutional policies or supervisory practices with certainty. This likely reflects a transitional situation in which both learners and supervisors are adapting to rapidly evolving GenAI tools and local guidance, as suggested by recent work by Blanco et al [45] and McCoy et al [46]. Fourth, the cross-sectional design provides a snapshot at a specific time point; given the rapid evolution of GenAI tools and institutional policies, the stability of these findings over time cannot be assumed. Fifth, interprofessional heterogeneity is an additional limitation: some health professions were underrepresented, limiting robust profession-specific comparisons. This reflects our primary aim of mapping placement-related practices rather than comparing disciplines; nonetheless, profession-specific differences in AI literacy and attitudes have been reported elsewhere and warrant dedicated analyses [47,48]. Finally, our GenAI maturity score should be considered an exploratory composite index of self-perceived readiness. Although internal consistency was good (Cronbach *α*=0.869), we did not undertake a full psychometric validation; further work should confirm and refine this construct.

### Perspectives

Future research should move beyond single institutions to include multicenter and international comparisons, enabling the identification of contextual variations and common challenges. Longitudinal designs will also be needed to capture how perceptions, competencies, and professional identity evolve as GenAI becomes more embedded in health professions education. Finally, triangulating student perspectives with those of supervisors, educators, and institutional leaders will be crucial to developing evidence-based strategies that responsibly integrate GenAI into curricula while respecting profession-specific needs.

### Conclusion

In conclusion, GenAI is already being used by French students in health professions during clinical placements, primarily as a cognitive and organizational aid. At the same time, students report concerns about dependence, skill erosion, and confidentiality, and express a strong demand for structured guidance. These findings support the need for explicit, ethically grounded, and supervised frameworks that align curricula, clinical supervision, and host-site governance so that GenAI use fosters reflexivity, autonomy, and professional accountability rather than unsupervised experimentation. More broadly, this echoes developments already observed across higher education and health care governance, including emerging institutional policies [13], the timely need for clear ethical and regulatory frameworks [49], and methodological approaches for developing evidence-based interdisciplinary guidelines [50].

## Supplementary material

10.2196/85243 Multimedia Appendix 1 Full survey questionnaire (French version with English translation) and supplementary tables (Tables S1-S11) reporting the generative artificial intelligence maturity items and detailed response distributions across key study domains.

10.2196/85243 Checklist 1 CHERRIES checklist for the web-based survey reporting.

## Abbreviations

AI: artificial intelligence
CHERRIES: Checklist for Reporting Results of Internet E-Surveys
GenAI: generative artificial intelligence
UGA: Université Grenoble Alpes
