# Supplementary material for: Health Professional Students’ Use of Generative Artificial Intelligence During Clinical Placements: Cross-Sectional Online Survey Study
Source: JMIR Med Educ. 2026 Apr 27;12:e85243. doi: 10.2196/85243 (PMC13120531; doi:10.2196/85243)
Supplement: Multimedia Appendix 1 [file mededu-v12-e85243-s001.docx]

**Table S1. Full survey questionnaire in French (*English translation*)**

| Questions | Pre-defined answer options | Types |
| --- | --- | --- |
| Quel est votre genre ?  *(What is your gender?)* | - Féminin *(Female)* - Masculin *(Male)* - Préfère ne pas répondre *(Preferred not to disclose)* - Autre *(Other)* | SCQ |
| Quelle est votre tranche d’âge ?  *(What is your age group?)* | - Moins de 18 ans *(< 18)* - 18-19 ans *(18-19)* - 20–24 ans *(20-24)* - 25–29 ans *(25-29)* - 30-34 ans *(30-34)* - 35 ans ou plus *(> 35)* | SCQ |
| Dans quelle filière de formation en santé êtes-vous inscrit(e) ?  *(In which health training program are you enrolled?)* | • Aides-soignants *(Nursing assistants)*  • Auxiliaires de puériculture *(Childcare assistants)*  • Kinésithérapie *(Physiotherapy)*   - Maïeutique *(Midwifery)* - Manipulateur radio *(Radio Manipulator)* - Médecine *(Medicine)* - Pharmacie *(Pharmacy)* - Préparateur en pharmacie *(Pharmacy technician)* - Soin infirmier *(Nursing)* - Odontologie *(Dentistry)* - Autre *(Other)* | SCQ |
| En quelle année de formation en santé êtes-vous inscrit(e) ?  *(In which health training program are you enrolled?)* | - 1ère année *(1st year)* - 2nde année *(2nd year)* - 3ème année *(3rd year)* - 4ème année *(4th year)* - 5ème année *(5th year)* - 6ème année *(6th year)* - Au-delà de la 6ème année *(> 6th year)* - Autre *(Other)* | SCQ |
| Avez-vous déjà utilisé une IAG dans le cadre d’un stage professionnel (externat, internat, stage clinique, etc.) ?  *(Have you ever used a generative AI tool during a professional placement or internship?)* | - Oui *(Yes)* - Non *(No)* | SCQ |
| Quelles sont les raisons pour lesquelles vous n’utilisez pas l’IAG dans votre pratique clinique ?  *(What are the reasons you do not use generative AI in your clinical practice?)* | - Je ne connaissais pas *(I didn't know)* - Cela ne m’intéresse pas *(I'm not interested in that)* - Je ne sais pas m’en servir *(I don't know how to use it)* - Je n’en ai pas besoin *(I don't need it)* - Je ne connais pas les bonnes pratiques pour l'utiliser *(I don't know the best practices for using it)* - Autre *(Other)* | SCQ |
| Quel(s) outil(s) d’IAG avez-vous utilisé dans le cadre de votre pratique clinique ?  *(Which generative AI tool(s) have you used in your clinical practice?)* | - ChatGPT - OpenAI - Gemini - Google - Copilot - Microsoft - Le Chat - Mistral - Claude AI - Anthropic - Llama - Meta - DeepSeek - DeepSeek - Dall-E - OpenAI - HuggingChat - HuggingFace - IAG sans internet *(GenAI offline)* - Autre (Other) | MCQ |
| Comment utilisez-vous ou avez-vous utilisé l'IA Générative dans votre pratique clinique pour traduire un texte écrit dans une autre langue ?  *(How do/did you use generative AI in your clinical practice to translate a text written in another language?)* | - Jamais *(Never)* - Rarement (Rarely) - Tous les mois *(Monthly)* - Toutes les semaines *(Weekly)* - Tous les jours *(Daily)* | SCQ |
| Comment utilisez-vous ou avez-vous utilisé l'IA Générative dans votre pratique clinique pour rédiger un rapport de stage (bilan, mémoires, etc..) ou un article scientifique ?  *(How do/did you use generative AI in your clinical practice to write a placement report (evaluation, thesis, etc.) or a scientific paper?)* | - Jamais *(Never)* - Rarement (Rarely) - Tous les mois *(Monthly)* - Toutes les semaines *(Weekly)* - Tous les jours *(Daily)* | SCQ |
| Comment utilisez-vous ou avez-vous utilisé l'IA Générative dans votre pratique clinique pour chercher des informations dans un texte (compte-rendu, article, cours, etc…) ?  *(How do/did you use generative AI in your clinical practice to extract information from a text (report, article, course material, etc.)?)* | - Jamais *(Never)* - Rarement (Rarely) - Tous les mois *(Monthly)* - Toutes les semaines *(Weekly)* - Tous les jours *(Daily)* | SCQ |
| Comment utilisez-vous ou avez-vous utilisé l'IA Générative dans votre pratique clinique pour effectuer une recherche bibliographique ?  *(How do/did you use generative AI in your clinical practice to perform a literature search?)* | - Jamais *(Never)* - Rarement (Rarely) - Tous les mois *(Monthly)* - Toutes les semaines *(Weekly)* - Tous les jours *(Daily)* | SCQ |
| Comment utilisez-vous ou avez-vous utilisé l'IA Générative dans votre pratique clinique pour simuler ou recréer une situation clinique ?  *(How do/did you use generative AI in your clinical practice to simulate or recreate a clinical situation?)* | - Jamais *(Never)* - Rarement (Rarely) - Tous les mois *(Monthly)* - Toutes les semaines *(Weekly)* - Tous les jours *(Daily)* | SCQ |
| Comment utilisez-vous ou avez-vous utilisé l'IA Générative dans votre pratique clinique pour rédiger un document concernant un patient ?  *(How do/did you use generative AI in your clinical practice to draft a document concerning a patient?)* | - Jamais *(Never)* - Rarement (Rarely) - Tous les mois *(Monthly)* - Toutes les semaines *(Weekly)* - Tous les jours *(Daily)* | SCQ |
| Comment utilisez-vous ou avez-vous utilisé l'IA Générative dans votre pratique clinique pour préparer une communication ou un entretien avec un patient ?  *(How do/did you use generative AI in your clinical practice to prepare communication or an interview with a patient?)* | - Jamais *(Never)* - Rarement (Rarely) - Tous les mois *(Monthly)* - Toutes les semaines *(Weekly)* - Tous les jours *(Daily)* | SCQ |
| Pour quelle(s) autre(s) tâche(s) avez-vous utilisé une IAG au cours d'un stage clinique ?  (*For what other tasks have you used generative AI during a clinical placement?)* | - | OQ |
| Avez-vous déjà mentionné une information personnelle réelle sensible comme un nom, un prénom, un numéro d’identification, une adresse complète, etc.. lors de l’utilisation d’une IAG ?  *(Have you ever entered sensitive personal information (e.g., name, ID number, full address) when using a generative AI tool?)* | - Cela ne m'est jamais arrivé *(This has never happened to me)* - Cela m'est arrivé une seule fois *(It has only happened to me once)* - Cela m'est arrivé quelques fois *(This has happened to me a few times)* - Cela m'arrive souvent *(This happens to me often)* - Cela m'est peut-être arrivé involontairement ; par exemple en faisant un copier-coller *(It may have happened to me unintentionally, e.g. by copying and pasting)* - Je ne sais pas si cela m'est arrivé *(I don't know if this has happened to me)* | SCQ |
| Avez-vous déjà mentionné une information personnelle sensible vous concernant comme votre nom, votre prénom, etc.. lors de l’utilisation d’une IAG ?  *(Have you ever entered sensitive personal information about yourself (e.g., name, first name) when using a generative AI tool?)* | - Cela ne m'est jamais arrivé *(This has never happened to me)* - Cela m'est arrivé une seule fois *(It has only happened to me once)* - Cela m'est arrivé quelques fois *(This has happened to me a few times)* - Cela m'arrive souvent *(This happens to me often)* - Cela m'est peut-être arrivé involontairement ; par exemple en faisant un copier-coller *(It may have happened to me unintentionally, e.g. by copying and pasting)* - Je ne sais pas si cela m'est arrivé *(I don't know if this has happened to me)* | SCQ |
| Avez-vous déjà utilisé une IAG pour traiter des données médicales réelles y compris de manière anonyme ?  *(Have you ever used a generative AI tool to process real medical data, even in anonymized form?)* | - Cela ne m'est jamais arrivé *(This has never happened to me)* - Cela m'est arrivé une seule fois *(It has only happened to me once)* - Cela m'est arrivé quelques fois *(This has happened to me a few times)* - Cela m'arrive souvent *(This happens to me often)* - Cela m'est peut-être arrivé involontairement ; par exemple en faisant un copier-coller *(It may have happened to me unintentionally, e.g. by copying and pasting)* - Je ne sais pas si cela m'est arrivé *(I don't know if this has happened to me)* | SCQ |
| Je pense que l'utilisation de l'IAG respecte les principes éthiques.  *(I believe the use of generative AI respects ethical principles.)* | - Pas du tout d'accord *(Strongly disagree)* - Pas d'accord *(Disagree)* - D'accord *(Agree)* - Tout à fait d'accord *(Strongly Agree)* - Je ne sais pas *(I don't know)* | LQ |
| Je pense que l'utilisation de l'IAG est suffisamment encadrée.  *(I believe the use of generative AI is sufficiently regulated.)* | - Pas du tout d'accord *(Strongly disagree)* - Pas d'accord *(Disagree)* - D'accord *(Agree)* - Tout à fait d'accord *(Strongly Agree)* - Je ne sais pas *(I don't know)* | LQ |
| Je pense que le personnel est formé à l'utilisation de l’IAG dans le contexte clinique.  *(I believe staff are trained to use generative AI in the clinical context.)* | - Pas du tout d'accord *(Strongly disagree)* - Pas d'accord *(Disagree)* - D'accord *(Agree)* - Tout à fait d'accord *(Strongly Agree)* - Je ne sais pas *(I don't know)* | LQ |
| Je pense que les biais des outils d’IAG sont pris en compte lors de son utilisation dans le contexte clinique.  *(I believe the biases of generative AI tools are taken into account when used in the clinical context.)* | - Pas du tout d'accord *(Strongly disagree)* - Pas d'accord *(Disagree)* - D'accord *(Agree)* - Tout à fait d'accord *(Strongly Agree)* - Je ne sais pas *(I don't know)* | LQ |
| Je pense que les patients doivent être informés lorsqu'une IAG est utilisée dans leur prise en charge.  *(I believe patients should be informed when a generative AI tool is used in their care.)* | - Pas du tout d'accord *(Strongly disagree)* - Pas d'accord *(Disagree)* - D'accord *(Agree)* - Tout à fait d'accord *(Strongly Agree)* - Je ne sais pas *(I don't know)* | LQ |
| Je considère que mes responsables ou encadrants de stage m’ont suffisamment informé(e) des conditions d’utilisation des IAG en stage clinique.  *(I believe my placement supervisors have sufficiently informed me about the conditions of generative AI use.)* | - Pas du tout d'accord *(Strongly disagree)* - Pas d'accord *(Disagree)* - D'accord *(Agree)* - Tout à fait d'accord *(Strongly Agree)* - Je ne sais pas *(I don't know)* | LQ |
| Je considère que mes responsables ou encadrants de stage m’ont suffisamment informé(e) des responsabilités légales si j'utilise une IAG en stage clinique.  *(I believe my placement supervisors have sufficiently informed me about the legal responsibilities associated with the use of generative AI.)* | - Pas du tout d'accord *(Strongly disagree)* - Pas d'accord *(Disagree)* - D'accord *(Agree)* - Tout à fait d'accord *(Strongly Agree)* - Je ne sais pas *(I don't know)* | LQ |
| En situation clinique, l’IAG pourrait permettre de faciliter la rédaction de documents cliniques.  *(In clinical settings, generative AI could facilitate drafting clinical documents.)* | - Pas du tout d'accord *(Strongly disagree)* - Pas d'accord *(Disagree)* - D'accord *(Agree)* - Tout à fait d'accord *(Strongly Agree)* | LQ |
| En situation clinique, l’IAG pourrait permettre d’améliorer la collecte d’informations patient.  *(In clinical settings, generative AI could improve patient information collection.)* | - Pas du tout d'accord *(Strongly disagree)* - Pas d'accord *(Disagree)* - D'accord *(Agree)* - Tout à fait d'accord *(Strongly Agree)* | LQ |
| En situation clinique, l’IAG pourrait permettre d’améliorer l’accès aux informations.  *(In clinical settings, generative AI could improve access to information.)* | - Pas du tout d'accord *(Strongly disagree)* - Pas d'accord *(Disagree)* - D'accord *(Agree)* - Tout à fait d'accord *(Strongly Agree)* | LQ |
| En situation clinique, l’IAG pourrait permettre de renforcer la précision des diagnostics.  *(In clinical settings, generative AI could increase diagnostic accuracy.)* | - Pas du tout d'accord *(Strongly disagree)* - Pas d'accord *(Disagree)* - D'accord *(Agree)* - Tout à fait d'accord *(Strongly Agree)* | LQ |
| En situation clinique, l’IAG pourrait permettre d’aider à créer des plans de soins personnalisés.  *(In clinical settings, generative AI could help create personalized care plans.)* | - Pas du tout d'accord *(Strongly disagree)* - Pas d'accord *(Disagree)* - D'accord *(Agree)* - Tout à fait d'accord *(Strongly Agree)* | LQ |
| En situation clinique, l’IAG pourrait permettre d’améliorer l’exactitude des pronostics.  *(In clinical settings, generative AI could improve prognostic accuracy.)* | - Pas du tout d'accord *(Strongly disagree)* - Pas d'accord *(Disagree)* - D'accord *(Agree)* - Tout à fait d'accord *(Strongly Agree)* | LQ |
| En situation clinique, l’IAG pourrait permettre d’augmenter l’efficacité des soins*.*  *(In clinical settings, generative AI could increase care efficiency.)* | - Pas du tout d'accord *(Strongly disagree)* - Pas d'accord *(Disagree)* - D'accord *(Agree)* - Tout à fait d'accord *(Strongly Agree)* | LQ |
| Avez-vous d’autres propositions concernant les potentielles utilisations de l’IAG en contexte clinique ?  *(Do you have other suggestions regarding potential uses of generative AI?)* | - | OQ |
| L’utilisation clinique de l’IAG pourrait créer une dépendance aux intelligences artificielles génératives.  *(The clinical use of generative AI could create dependence.)* | - Pas du tout d'accord *(Strongly disagree)* - Pas d'accord *(Disagree)* - D'accord *(Agree)* - Tout à fait d'accord *(Strongly Agree)* | LQ |
| L’utilisation clinique de l’IAG pourrait contribuer à une perte de compétences cliniques à long terme.  *(The clinical use of generative AI could contribute to long-term loss of clinical skills.)* | - Pas du tout d'accord *(Strongly disagree)* - Pas d'accord *(Disagree)* - D'accord *(Agree)* - Tout à fait d'accord *(Strongly Agree)* | LQ |
| L’utilisation clinique de l’IAG pourrait remplacer le personnel soignant ou des spécialités.  *(The clinical use of generative AI could replace healthcare staff or certain specialties.)* | - Pas du tout d'accord *(Strongly disagree)* - Pas d'accord *(Disagree)* - D'accord *(Agree)* - Tout à fait d'accord *(Strongly Agree)* | LQ |
| L’utilisation clinique de l’IAG pourrait augmenter le temps de formation des soignants.  *(The clinical use of generative AI could lengthen healthcare training programs.)* | - Pas du tout d'accord *(Strongly disagree)* - Pas d'accord *(Disagree)* - D'accord *(Agree)* - Tout à fait d'accord *(Strongly Agree)* | LQ |
| L’utilisation clinique de l’IAG pourrait remettre en question les compétences professionnelles.  *(The clinical use of generative AI could challenge professional competencies.)* | - Pas du tout d'accord *(Strongly disagree)* - Pas d'accord *(Disagree)* - D'accord *(Agree)* - Tout à fait d'accord *(Strongly Agree)* | LQ |
| L’utilisation clinique de l’IAG pourrait augmenter le risque de violation du secret médical.  *(The clinical use of generative AI could increase the risk of breaching medical confidentiality.)* | - Pas du tout d'accord *(Strongly disagree)* - Pas d'accord *(Disagree)* - D'accord *(Agree)* - Tout à fait d'accord *(Strongly Agree)* | LQ |
| L’utilisation clinique de l’IAG pourrait augmenter les inégalités d’accès aux soins.  *(The clinical use of generative AI could increase inequalities in access to care.)* | - Pas du tout d'accord *(Strongly disagree)* - Pas d'accord *(Disagree)* - D'accord *(Agree)* - Tout à fait d'accord *(Strongly Agree)* | LQ |
| Avez-vous d’autres propositions concernant les risques liés à l'utilisations de l’IAG en contexte clinique ?  *(Do you have other suggestions regarding risks related to the use of generative AI?)* | - | OQ |
| Je me sens à l’aise avec les technologies numériques en général.  *(I feel comfortable with digital technologies in general.)* | - Pas du tout d'accord *(Strongly disagree)* - Pas d'accord *(Disagree)* - D'accord *(Agree)* - Tout à fait d'accord *(Strongly Agree)* | LQ |
| Avez-vous déjà suivi un cours ou une formation spécifique à l’intelligence artificielle ?  *(Have you ever attended a training course specifically on artificial intelligence?)* | - Oui *(Yes)* - Non *(No)* | SCQ |
| Ces cours ou formations ont-ils traités du sujet de l’IA générative ?  *(Did this training address generative AI?)* | - Oui *(Yes)* - Non *(No)* | SCQ |
| J'ai trouvé cette formation adaptée pour savoir comment utiliser efficacement l’IAG dans mon activité professionnelle ?  *(I found this training appropriate to learn how to use generative AI effectively.)* | - Pas du tout d'accord *(Strongly disagree)* - Pas d'accord *(Disagree)* - D'accord *(Agree)* - Tout à fait d'accord *(Strongly Agree)* | LQ |
| Je comprends les concepts de base de l’IAG.  *(I understand the basic concepts of generative AI.)* | - Pas du tout d'accord *(Strongly disagree)* - Pas d'accord *(Disagree)* - D'accord *(Agree)* - Tout à fait d'accord *(Strongly Agree)* | LQ |
| Toutes les IA ne sont pas des IAG.  *(Not all AI systems are generative AI.)* | - Pas du tout d'accord *(Strongly disagree)* - Pas d'accord *(Disagree)* - D'accord *(Agree)* - Tout à fait d'accord *(Strongly Agree)* | LQ |
| Je sais évaluer la fiabilité d’une réponse fournie par une IAG.  *(I know how to assess the reliability of a response provided by generative AI.)* | - Pas du tout d'accord *(Strongly disagree)* - Pas d'accord *(Disagree)* - D'accord *(Agree)* - Tout à fait d'accord *(Strongly Agree)* | LQ |
| Je sais dans quels contextes je peux utiliser une IAG.  *(I know in which contexts I can use generative AI.)* | - Pas du tout d'accord *(Strongly disagree)* - Pas d'accord *(Disagree)* - D'accord *(Agree)* - Tout à fait d'accord *(Strongly Agree)* | LQ |
| Je connais les limites actuelles des modèles d’IAG*.*  *(I am aware of the current limitations of generative AI models.)* | - Pas du tout d'accord *(Strongly disagree)* - Pas d'accord *(Disagree)* - D'accord *(Agree)* - Tout à fait d'accord *(Strongly Agree)* | LQ |
| Je sais où trouver des ressources fiables pour me former à l’IAG.  *(I know where to find reliable resources to learn about generative AI.)* | - Pas du tout d'accord *(Strongly disagree)* - Pas d'accord *(Disagree)* - D'accord *(Agree)* - Tout à fait d'accord *(Strongly Agree)* | LQ |
| Je sais comment utiliser l’IAG dans ma formation professionnelle.  *(I know how to use generative AI in my professional training.)* | - Pas du tout d'accord *(Strongly disagree)* - Pas d'accord *(Disagree)* - D'accord *(Agree)* - Tout à fait d'accord *(Strongly Agree)* | LQ |
| Je sais comment utiliser l’IAG dans ma pratique professionnelle.  *(I know how to use generative AI in my professional practice.)* | - Pas du tout d'accord *(Strongly disagree)* - Pas d'accord *(Disagree)* - D'accord *(Agree)* - Tout à fait d'accord *(Strongly Agree)* | LQ |
| J’ai besoin d’une formation théorique sur les IAG.  *(I need theoretical training on generative AI.)* | - Pas du tout d'accord *(Strongly disagree)* - Pas d'accord *(Disagree)* - D'accord *(Agree)* - Tout à fait d'accord *(Strongly Agree)* | LQ |
| J’ai besoin d’une formation pratique sur les IAG.  *(I need practical training on generative AI.)* | - Pas du tout d'accord *(Strongly disagree)* - Pas d'accord *(Disagree)* - D'accord *(Agree)* - Tout à fait d'accord *(Strongly Agree)* | LQ |
| J’ai besoin d’une formation sur la collaboration Humain–IA.  *(I need training on Human–AI collaboration.)* | - Pas du tout d'accord *(Strongly disagree)* - Pas d'accord *(Disagree)* - D'accord *(Agree)* - Tout à fait d'accord *(Strongly Agree)* | LQ |
| J’ai besoin d’une formation de sensibilisation aux enjeux éthiques, réglementaires et déontologiques.  *(I need training on ethical, regulatory, and professional issues related to generative AI.)* | - Pas du tout d'accord *(Strongly disagree)* - Pas d'accord *(Disagree)* - D'accord *(Agree)* - Tout à fait d'accord *(Strongly Agree)* | LQ |
| J’ai besoin d’un accompagnement sur l’utilisation des IAG qui soit personnalisé et adapté à ma profession de santé.  *(I need personalized support tailored to my health profession.)* | - Pas du tout d'accord *(Strongly disagree)* - Pas d'accord *(Disagree)* - D'accord *(Agree)* - Tout à fait d'accord *(Strongly Agree)* | LQ |
| J’ai besoin d’accéder à une plateforme de ressources d’autoformation (MOOC, tutoriels, FAQ).  *(I need access to a self-learning platform (MOOC, tutorials, FAQ).)* | - Pas du tout d'accord *(Strongly disagree)* - Pas d'accord *(Disagree)* - D'accord *(Agree)* - Tout à fait d'accord *(Strongly Agree)* | LQ |
| J’ai besoin d’avoir un guide de recommandation du bon usage des IAG dans la pratique clinique.  *(I need a best-practice guide for the clinical use of generative AI.)* | - Pas du tout d'accord *(Strongly disagree)* - Pas d'accord *(Disagree)* - D'accord *(Agree)* - Tout à fait d'accord *(Strongly Agree)* | LQ |
| Quels sont vos autres besoins de formation, supports ou accompagnement sur les IAG ?  *(What other training or support needs do you have regarding generative AI?)* | - | OQ |

**SCQ: Single Choice Question; MCQ: Multiple Choice Question ; LQ : Linkert Question ; OQ : Open Question**

**Table S2. Self-reported GenAI maturity (N=380 valid responses ^a^)**

a. Distribution of the GenAI maturity index

| Score Level | n (%) |
| --- | --- |
| Minimal (1) | 22 (5.8) |
| Limited (2) | 130 (34.2) |
| Moderate (3) | 199 (52.4) |
| High (4) | 29 (7.6) |

^a^ Valid responses = respondents with ≥5 non-missing answers out of 9 items

b. Knowledge and self-efficacy regarding GenAI (NA excluded)

| Item | Strongly Disagree  n (%)  1 | Disagree  n (%)  2 | Agree  n (%)  3 | Strongly Agree  n (%)  4 | Median [IQR]^a^ |
| --- | --- | --- | --- | --- | --- |
| I Feel Comfortable With Digital Technologies | 18  (4.6) | 64  (16.5) | 207  (53.4) | 99  (25.5) | Agree (3)  [3–4] |
| I Understand The Basics Of Generative AI | 16  (4.3) | 63  (16.8) | 228  (61.0) | 67  (17.9) | Agree (3)  [3–3] |
| Not All AI Is Generative AI | 11  (3.1) | 41  (11.4) | 223  (62.1) | 84  (23.4) | Agree (3)  [3–3] |
| I Can Assess The Reliability Of A Generative AI Answer | 31  (8.1) | 117  (30.7) | 186  (48.8) | 47  (12.3) | Agree (3)  [2–3] |
| I Know The Contexts Where I Can Use Generative AI | 28  (7.5) | 103  (27.4) | 180  (47.9) | 65  (17.3) | Agree (3)  [2–3] |
| I Know The Current Limitations Of Generative AI Models | 40  (10.6) | 149  (39.6) | 130  (34.6) | 57  (15.2) | Disagree (2)  [2–3] |
| I Know Where To Find Reliable Resources To Train On Generative AI | 108  (28.6) | 199  (52.7) | 55  (14.6) | 16  (4.2) | Disagree (2)  [1–2] |
| I Know How To Use Generative AI In My Academic Training | 50  (13.3) | 125  (33.2) | 163  (43.2) | 39  (10.3) | Agree (3)  [2–3] |
| I Know How To Use Generative AI In My Professional Practice | 72  (19.2) | 146  (38.8) | 131  (34.8) | 27  (7.2) | Disagree (2)  [2–3] |

^a^ Median and interquartile range of the 4-point Likert response (1 = strongly disagree, 4 = strongly agree)

**Table S3. Additional self-reported reasons for not adopting GenAI (N=25 responses; 29 meaning units)**

| Subtheme | Operational definition | Meaning units n (%) | Illustrative quote  english translated |
| --- | --- | --- | --- |
| Reliability / errors | Non-use framed as mistrust in accuracy or risk of errors | 5  (17) | “I do not find it reliable.” |
| Environmental impact / socio-technical critique | Ecological footprint, resource use, dependence on big tech, exploitation concerns | 5  (17) | “An extremely polluting tool” ;“Increasing our dependence on big tech.” |
| Preference for official sources / protocols / staff advice | Reliance on protocols, official websites, or supervisors instead of GenAI | 4  (14) | “I rely on service protocols and on staff advice.” |
| Reasoning/relational concerns | Fear of reduced reflective capacity, depersonalization | 2  (7) | “Alteration of reflective capacity and a form of depersonalization.” |
| Other low-frequency subthemes | Confidentiality, opposition in principle, fear of judgement, lack of opportunity/salience | 13  (45) |  |

**Table S4. Additional self-reported uses of GenAI (N=116 responses; 106 meaning units)**

| Subtheme | Operational definition | Meaning units n (%) | Illustrative quote  english translated |
| --- | --- | --- | --- |
| Writing / text revision | Spelling, grammar, reformulation, email/report wording | 24  (22.6) | “Spelling correction.” |
| Learning support / explanations | Definitions, explanations, understanding concepts/pathophysiology | 25  (23.6) | “Help me understand certain things.” |
| Medication-related queries | Posology, interactions, adverse effects, contraindications, iatrogeny | 13  (12.3) | “Every month, to check adverse effects or interactions between medications.” |
| Internship organization | Internship objectives, assignments, oral preparation, applications, protocols | 12  (11.3) | “Format my internship objectives.” |
| Clinical reasoning support | Clinical approach, hypotheses, analysis of situations (non-medication-focused) | 8  (7.5) | “Before a clinical exam, I sometimes ask it to explain which avenues to explore.” |
| Synthesis / summaries | Summary sheets, recaps, structured syntheses | 7  (6.6) | “Summary sheets on diseases or medications.” |
| Literature search / pubmed | PubMed queries, finding articles, search strategy optimization | 4  (3.8) | “Correcting and optimizing PubMed search strings (rarely).” |
| Learning materials (slides/schematics) | PowerPoint, schematics, clearer visuals | 3  (2.8) | “Creating PowerPoint slides.” |
| Coding / data assistance | R/SAS/VBA/Excel/statistics support | 3  (2.8) | “Help with coding (SAS, R).” |
| Translation | Content translation | 1  (0.9) | “French–English translation (once).” |
| Scientific news monitoring | Updates/news summaries | 1  (0.9) | “Summaries of recent news and research.” |
| Clinical imaging/visuals | Help with interpreting or reviewing imaging examples | 1  (0.9) | “Examples of imaging that are difficult to interpret.” |
| Transcription/anamnesis | Dictation to written anamnesis | 1  (0.9) | “Writing the anamnesis from voice dictation.” |
| Ethical dilemma discussion | Reflection on ethical dilemmas | 1  (0.9) | “Analysis of ethical dilemmas.” |
| Out-of-scope personal use | Non-internship/personal use | 1  (0.9) | “Sports program.” |
| Patient communication support | Simplifying explanations for patients, destigmatizing, phrasing | 1  (0.9) | “Helping me explain a medical consultation in accessible terms (eg, destigmatizing antidepressant treatment, finding a better phrasing to explain a ‘simple’ diagnosis).” |

**Table S5. Perceived Benefits of GenAI in Clinical Practice (N=388)**

| Perceived Benefit | Strongly Disagree  n (%)  1 | Disagree  n (%)  2 | Agree  n (%)  3 | Strongly Agree  n (%)  4 | Median [IQR]^a^ |
| --- | --- | --- | --- | --- | --- |
| Facilitate drafting of clinical documents | 28  (7.2) | 45  (11.6) | 230  (59.3) | 85  (21.9) | Agree (3)  [3-3] |
| Improve patient-information collection | 81  (20.9) | 131  (33.7) | 135  (34.8) | 41  (10.6) | Disagree (2)  [2-3] |
| Improve information accessibility | 44  (11.3) | 78  (20.1) | 201  (51.8) | 65  (16.7) | Agree (3)  [2-3] |
| Increase diagnostic accuracy | 57  (14.7) | 132  (34.0) | 158  (40.7) | 41  (10.6) | Agree (3)  [2-3] |
| Support personalised care-plan creation | 50  (12.9) | 87  (22.4) | 203  (52.3) | 48  (12.4) | Agree (3)  [2-3] |
| Improve prognostic accuracy | 81  (20.9) | 153  (39.4) | 122  (31.4) | 32  (8.2) | Disagree (2)  [2-3] |
| Increase care efficiency | 63  (16.2) | 116  (29.9) | 172  (44.3) | 37  (9.5) | Agree (3)  [2-3] |

^a^ Median category and inter-quartile range (Q1 – Q3) on the 4-point Likert scale.

**Table S6. Additional self-reported proposals on potential clinical uses of GenAI ( N=26 responses; 22 meanings units)**

| Subtheme | Operational definition | Meaning units,  n (%) | Illustrative quote  english translated |
| --- | --- | --- | --- |
| Reflective support / checklists / red flags | Systematization, “not forgetting” items, red flags | 4  (18.2) | “To reassure myself that I’ve ‘thought of everything’ “; “red flags” |
| Administrative/documentation streamlining | Certificates, letters, documentation, traceability | 3  (13.6) | “Reducing administrative burden” |
| Clinical reasoning / differential diagnosis | Exploring hypotheses while emphasizing verification and limits | 3  (13.6) | “To make sure I haven’t missed an etiology” |
| Patient communication / plain language | Explaining diagnoses with accessible wording | 2  (9.1) | “Explaining certain diagnoses in more accessible terms” |
| Refusal / no proposal | Explicit refusal or “no” | 2  (9.1) | “No” |
| Access constraints | Unequal access depending on subscription | 2  (9.1) | “Paid and free versions differ”; “access is not equal for everyone” |
| Transcription / interview | Transcribing patient interviews | 1  (4.5) | “Transcription of the patient interview” |
| Scientific monitoring | New studies, updates | 1  (4.5) | “Keeping up to date with newly published studies” |
| Medication safety/iatrogeny prevention | Preventing medication-related harm | 1  (4.5) | “Preventing iatrogeny” |
| Student support for care delivery | Student-facing checklists for essential care steps | 1  (4.5) | “Generating a recap sheet so students don’t forget key examinations” |
| Radiology support with human review | Flagging findings with mandatory clinician validation | 1  (4.5) | “Used in radiology to flag potential fractures, with physician review” |
| Service organization | Workflow/service organization | 1  (4.5) | “Service organization” |

**Table S7. Perceived risks of clinical use of GenAI (N=388)**

| Perceived Risk | Strongly Disagree  n (%)  1 | Disagree  n (%)  2 | Agree  n (%)  3 | Strongly Agree  n (%)  4 | Median  [IQR] ^a^ |
| --- | --- | --- | --- | --- | --- |
| Create dependency on AI | 3  (0.8) | 32  (8.3) | 185  (47.7) | 168  (43.3) | Agree (3)  [3-4] |
| Long-term loss of clinical skills | 8  (2.1) | 51  (13.1) | 153  (39.4) | 176  (45.4) | Agree (3)  [3-4] |
| Replace healthcare staff or specialties | 92  (23.7) | 151  (38.9) | 89  (22.9) | 56  (14.4) | Disagree (2)  [2-3] |
| Increase training time for caregivers | 45  (11.6) | 205  (52.8) | 118  (30.4) | 20  (5.2) | Disagree (2)  [2-3] |
| Challenge professional competencies | 42  (10.8) | 118  (30.4) | 153  (39.4) | 75  (19.3) | Agree (3)  [2-3] |
| Raise risk of breaching medical confidentiality | 6  (1.6) | 43  (11.1) | 155  (40.0) | 184  (47.4) | Agree (3)  [3-4] |
| Exacerbate inequalities of access to care | 55  (14.2) | 186  (47.9) | 85  (21.9) | 62  (16.0) | Disagree (2)  [2-3] |

^a^ Median category and inter-quartile range (Q1 – Q3) on the 4-point Likert scale.

**Table S8. Additional self-reported proposals on potential risks of clinical use of GenAI (N=32 responses; 32 meanings units)**

| Subtheme | Operational definition | Meaning units,  n (%) | Illustrative quote  english translated |
| --- | --- | --- | --- |
| Errors / patient safety | Inaccurate outputs, potential harm, poor management | 6  (19) | “Inaccuracy in the information provided or in generated clinical letters.” |
| Reduced reflection / dependence | Delegation of thinking, reduced critical reasoning, dependency | 5  (16) | “The main risk is to stop thinking and let the system do it.” |
| Relational degradation / depersonalization | Reduced quality of care relationship, depersonalization | 5  (16) | “A form of depersonalization.” |
| Confidentiality / data monetization | Breach of confidentiality, commercial reuse of health data | 4  (12) | “Risk of monetization of health data.” |
| Overreliance / confirmation bias | Excessive trust, false reassurance, confirmation of initial opinion | 3  (9) | “Excessive reassurance because the system confirms one’s opinion.” |
| Organizational impact | Reduced peer consultation, replacing human discussion | 2  (6) | “Fewer discussions between clinicians because people would ask GenAI instead.” |
| Responsibility dilution | Unclear accountability and professional responsibility | 1  (3.1) | “Forgetting who holds responsibility on the clinician’s side.” |
| Non-verifiability / lack of sources | Outputs not auditable when not sourced | 1  (3) | “Results are not verifiable if they are not sourced.” |
| Patient misuse / self-medication | Patients acting on GenAI advice | 1  (3) | “Patients who think they can treat themselves based on what GenAI tells them.” |
| Broader sociotechnical fears | “Loss of control” narratives | 1  (3) | “The more we make it ‘intelligent,’ the more it will take control of what people think and see.” |
| Unclassifiable |  |  |  |

**Table S9. Stated training and support needs for GenAI use (NA excluded)**

| Need item | Strongly Disagree  n (%)  1 | Disagree  n (%)  2 | Agree  n (%)  3 | Strongly Agree  n (%)  4 | Median  [IQR] ^a^ |
| --- | --- | --- | --- | --- | --- |
| Theoretical training on Generative AI | 37  (9.9) | 100  (26.8) | 155  (41.5) | 81  (21.7) | Agree (3)  [2-3] |
| Practical, hands-on training | 39  (10.4) | 93  (24.8) | 151  (40.3) | 92  (24.5) | Agree (3)  [2-3] |
| Training in human–AI collaboration | 31  (8.3) | 71  (19.0) | 181  (48.5) | 90  (24.1) | Agree (3)  [2-3] |
| Ethics / regulatory awareness training | 26  (6.9) | 58  (15.3) | 171  (45.2) | 123  (32.5) | Agree (3)  [3-4] |
| Personalised, profession-specific coaching | 32  (8.6) | 65  (17.4) | 176  (47.0) | 101  (27.0) | Agree (3)  [2-4] |
| Access to self-learning platform (mooc, tutorials) | 38  (10.2) | 105  (28.3) | 157  (42.3) | 71  (19.1) | Agree (3)  [2-3] |
| Best-practice guide for clinical use | 33  (8.8) | 48  (12.9) | 190  (50.9) | 102  (27.3) | Agree (3)  [3-4] |

^a^ Median category and inter-quartile range (Q1 – Q3) on the 4-point Likert scale.

**Table S10. Additional self-reported proposals on training and support needs (N=27 responses; 24 meanings units)**

| Subtheme | Operational definition | Meaning units,  n (%) | Illustrative quote  english translated |
| --- | --- | --- | --- |
| No additional needs / no interest | Explicitly reports no need or no interest | 8  (33) | “No need—this does not interest me at all.” |
| Practical hands-on training / support | Workshops, practice, coaching, guidance to use appropriately | 6  (25) | “Practical training would interest me a lot—I know I haven’t optimized how I use it.” |
| Indeterminate | “No idea” / “don’t know” | 2  (8) | “No idea.” |
| Best-practice guide | Do/don’t recommendations, safe use guidance | 1  (4) | “I’d like a best-practice guide to avoid compromising confidentiality.” |
| Ethical foundations | Clarifying ethical basis and principles | 1  (4) | “We need to define what the ethics of GenAI should be, and where it comes from.” |
| Local/open-source tools | On-site hosting, open-source tools | 1  (4) | “A local, open-source tool hosted on site.” |
| Academic integrity/authorship | Rules for theses/articles, ownership/limits | 1  (4) | “For theses and papers: what is the limit—when can I use it, and does the output remain mine?” |
| Environmental impact | Training on ecological footprint | 1  (4) | “Raise awareness about the environmental impact of GenAI.” |
| In-person format | Preference for face-to-face training | 1  (4) | “In person.” |
| Curated trustworthy resources | Reliable resources, curated sources | 1  (4) | “Help finding reliable resources.” |
| Clarifying limits/contexts of use | When/where GenAI can be used | 1  (4) | “Limits of GenAI and appropriate domains of use?” |

**Table S11. Governance and organizational readiness (NA excluded)**

| Item | Strongly Disagree  n (%)  1 | Disagree  n (%)  2 | Agree  n (%)  3 | Strongly Agree  n (%)  4 | Median  [IQR] ^a^ |
| --- | --- | --- | --- | --- | --- |
| My supervisors informed me about the legal responsibilities | 219  (59.8) | 96  (26.2) | 41  (11.) | 10  (2.8) | Strongly disagree (1) [1–2] |
| My supervisors informed me about conditions of AI se during placements | 206  (56.7) | 101  (27.8) | 43  (11.8) | 13  (3.6) | Strongly disagree (1) [1–2] |
| I think generative-AI use respects ethical principles | 34  (11.0) | 159  (51.3) | 107  (34.5) | 10  (3.2) | Disagree (2) [2–3] |
| I think it is sufficiently regulated | 117  (32.6) | 206  (57.4) | 32  (8.9) | 4  (1.1) | Disagree (2) [1–2] |
| I think staff are trained for clinical use | 217  (58.3) | 137  (36.8) | 17  (4.6) | 1  (0.3) | Strongly disagree (1) [1–2] |
| Patients should be informed when AI is used | 16  (4.8) | 67  (20.) | 130  (39.3) | 118  (35.6) | Agree (3)  [2–4] |
| I think tool biases are accounted for | 99  (28.9) | 188  (54.8) | 50  (14.6) | 6  (1.7) | Disagree (2) [1–2] |

^a^ Median category and inter-quartile range (Q1 – Q3) on the 4-point Likert scale.

Table S12. Discipline-specific response rates (N=4,553)

| Disciplines | Eligible students, n | Respondents, n (%) |
| --- | --- | --- |
| Total | 4,553 | 388 (8.5%) |
| Medicine | 2196 | 69 (3.1) |
| Midwifery | 120 | 23 (19.1) |
| Nursing | 1904 | 217 (11.4) |
| Pharmacy | 283 | 66 (23.3) |
| Physiotherapy | 50 | 13 (26) |
