# Supplementary material for: Health Professional Students’ Use of Generative Artificial Intelligence During Clinical Placements: Cross-Sectional Online Survey Study
Source: JMIR Med Educ. 2026 Apr 27;12:e85243. doi: 10.2196/85243 (PMC13120531; doi:10.2196/85243)
Supplement: Checklist 1 [file mededu-v12-e85243-s002.docx]

**Table 1.**Checklist for Reporting Results of Internet E-Surveys (CHERRIES)

| 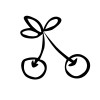 | **Checklist for Reporting Results of Internet E-Surveys (CHERRIES)** | |
| --- | --- | --- |
| ***Item Category*** | ***Checklist Item*** | ***Explanation*** |
| **Design** | Describe survey design | Cross-sectional, web-based survey; convenience sample of UGA health professional students (medicine, pharmacy, nursing, midwifery, physiotherapy) currently in, or within past 18 months of, a clinical placement. |
| **IRB (Institutional Review Board) approval and informed consent process** | IRB approval | Approved by “Committee for Integrity and Ethics in Research in Health Professions Education” (SIFEM); Notice n°1905-2025; July 17, 2025. |
|  | Informed consent | Electronic information sheet + e-consent before accessing the questionnaire; voluntariness, confidentiality, right to withdraw, purpose of study; expected duration ~15 min. |
|  | Data protection | No personally identifying information collected. Data access restricted to research team. |
| **Development and pre-testing** | Development and testing | Items adapted from prior studies; expert review by 2 clinicians/HPE specialists; pilot test with 10 students; technical functionality and intelligibility confirmed; average completion time ~15 min. |
| **Recruitment process and description of the sample having access to the questionnaire** | Open survey versus closed survey | Open survey (anonymous link; not password-protected), distributed to an enumerated sampling frame (UGA mailing lists) |
|  | Contact mode | Initial contact via official academic mailing lists (email); additional offline recruitment via printed flyers. |
|  | Advertising the survey | The survey invitation was disseminated via official university academic mailing lists targeting all eligible students across the participating programs, ensuring comprehensive outreach within the defined sampling frame. Printed flyers displayed at the entrances of lecture halls and classrooms served only as reminders during the recruitment period |
|  | Web/E-mail | Web-based questionnaire hosted on LimeSurvey; access via anonymous link; responses captured automatically in LimeSurvey database. |
| **Survey administration** | Context | The survey was not hosted/promoted on a thematic website or online community; it was delivered via LimeSurvey and disseminated through official university mailing lists. Hence, selection bias is primarily related to voluntary participation among invited students, not to the influence of website content. |
|  | Mandatory/voluntary | Voluntary survey; no requirement to access any website. |
|  | Incentives | No incentives offered. |
|  | Time/Date | June 1 to September 1, 2025. |
|  | Randomization of items or questionnaires | No randomization reported. |
|  | Adaptive questioning | Yes. A single skip-logic was implemented: participants reporting GenAI use during clinical placements were directed to follow-up questions on use (tools/tasks/frequency), while participants reporting no use were directed to questions on reasons for non-use. No additional branching was used. Questions on perceived benefits, risks, governance, and training/support needs were displayed to all participants.  This minimal branching was used to reduce respondent burden and avoid presenting irrelevant items while preserving a common core of perception and governance items across the full sample. |
|  | Number of Items | 61 total items (18 single-choice, 38 Likert single-choice, 1 multiple-choice, 4 open-ended). |
|  | Number of screens (pages) | The survey was distributed over 5 screens/pages: 1 introductory page (study information, data processing information, and electronic consent) followed by 4 questionnaire pages. |
|  | Completeness check | Yes, partially. A total of 38 items were mandatory and could not be left blank. For all other items, missing responses were allowed. No additional automated completeness/consistency checks were implemented beyond item-level mandatory constraints (ie, no global validation before final submission). |
|  | Review step | Yes. Respondents could review and change their answers before final submission (eg, using the Back function between pages). |
| **Response rates** |  |  |
|  | Unique site visitor | \| Not measurable in a robust way because the survey was distributed via email/offline and not embedded in a single website with stable traffic denominators. \| \| --- \|  \|  \| \| --- \| |
|  | View rate (Ratio of unique survey visitors/unique site visitors) | \| Not available (no reliable count of unique visitors landing on first page relative to site visitors). \| \| --- \|  \|  \| \| --- \| |
|  | Participation rate (Ratio of unique visitors who agreed to participate/unique first survey page visitors) | not available |
|  | Completion rate (Ratio of users who finished the survey/users who agreed to participate) | With a separate consent page, completion was defined as submitted questionnaires / consenting participants; 388/631 (61.5%) |
| **Preventing multiple entries from the same individual** |  |  |
|  | Cookies used | Not used. |
|  | IP check | Not used |
|  | Log file analysis | Not performed |
|  | Registration | Not applicable |
| **Analysis** |  |  |
|  | Handling of incomplete questionnaires | Only questionnaires submitted on the final page were analyzed; questionnaires that terminated early were excluded |
|  | Questionnaires submitted with an atypical timestamp | No completion-time cutoffs applied |
|  | Statistical correction | No weighting or propensity-score adjustments were applied to correct for potential non-representativeness of the convenience sample |
